# The Effect of Corticotomy-Assisted Orthodontic Therapy (CAOT) or Periodontally Accelerated Osteogenic Orthodontics (PAOO) on Bone Remodeling and the Health of Periodontium: A Systematic Review of Systematic Reviews

**DOI:** 10.3390/jcm13195726

**Published:** 2024-09-26

**Authors:** Anna Ewa Kuc, Maria Kulgawczyk, Magdalena Ewa Sulewska, Natalia Kuc, Beata Kawala, Joanna Lis, Michał Sarul, Jacek Kotuła

**Affiliations:** 1 Department of Dentofacial Orthopaedics and Orthodontics, Wroclaw Medical University, Krakowska 26, 50-425 Wroclaw, Poland; beata.kawala@umw.edu.pl (B.K.); joanna.lis@umed.wroc.pl (J.L.); j_kotula@poczta.onet.pl (J.K.); 2 Dental Star Specialist Center for Aesthetic Dentistry, ul. Konopnicka 1c/ U3, 15-215 Bialystok, Poland; kulgawczyk.m@gmail.com; 3 Department of Periodontal and Oral Mucosa Diseases, ul. Waszyngtona 13, 15-269 Bialystok, Poland; magdalena.sulewska@umb.edu.pl; 4 Faculty of Medicine, Medical University in Bialystok, ul. Kilińskiego 1, 15-089 Bialystok, Poland; nataliakuc.med@gmail.com; 5 Department of Integrated Dentistry, Wroclaw Medical University, 50-425 Wroclaw, Poland; michal.sarul@umw.edu.pl

**Keywords:** bone remodeling, corticotomy-assisted orthodontic therapy (CAOT), periodontally accelerated osteogenic orthodontics (PAOO), orthodontic tooth movement, periodontal ligament strain, fenestrations

## Abstract

**Background:** Orthodontic treatment involves moving teeth within the alveolar ridge. Bone remodeling is associated with the activity of osteoblasts and osteoclasts. Procedures such as corticotomy-assisted orthodontic therapy (CAOT) or periodontally accelerated osteogenic orthodontics (PAOO) are intended to reduce bone density and negative stress on the grip side and therefore limit bone resorption during orthodontic movement or add bone substitute material so that the tooth does not cross the vestibular plate. **Methods:** The study was conducted in accordance with the PRISMA (Preferred Reporting Items for Systematic Reviews and Meta-Analyses) guidelines. The study design was defined in the PICO format—Population (P): patients with full permanent dentition, both adolescents and adults; Intervention (I): orthodontic treatment with fixed appliances using additional supportive treatments such as CAOT or PAOO; Comparison (C): assessment of the impact of additional treatments during orthodontic treatment on the remodeling of the alveolar bone and the condition of the periodontium; Result (O): statistically significant/non-significant differences in the condition of the alveolar bone before and after orthodontic treatment. Search filters include the time of publication of the article, systematic reviews from the last five years, and publications that appeared in English. The information provided in the abstracts of systematic reviews that describe the effects of additional procedures during orthodontic treatment such as CAOT or PAOO on the health of periodontium was analyzed. Articles unrelated to the subject of the planned study and those in which tooth movement acceleration was analyzed were excluded. **Results:** Eight articles were selected in which a total number of 835 subjects took part. The changes in bone density and effects on periodontium were different after CAOT and PAOO. **Conclusions:** The validity of CAOT and PAOO procedures remains controversial. Better results are obtained when combined with tissue augmentation or thickening of the gingival phenotype rather than as stand-alone procedures, as their uses to protect periodontal tissues are limited.

## 1. Introduction

Orthodontic treatment involves moving teeth within the alveolar bone. The relief of crowding and the compensation or decompensation of skeletal or dental defects may lead to bone dehiscence or fenestration and gum recession due to exceeding the so-called bone envelope [1]. For years, attempts have been made to apply more/less invasive procedures to minimize the above risk.

In light of publications from recent years that precisely describe the biomechanics of orthodontic tooth movement (OTM), during which, because of the force applied on the pressure side, bone tissue is resorbed, and on the opposite side the tissue is layered, you can assume that such movement occurs through the bone, not with the bone, as it was once believed [2]. Such bone remodeling is essential for effective orthodontic treatment and understanding the remodeling processes is key to optimizing orthodontic treatment and minimizing side effects [3].

The first reaction of the tooth to the applied orthodontic force is displacement within the periodontal ligaments, causing deformation and stresses in the periodontal ligament (PDL). There is a negative tension on the pressure side and a positive tension on the pull side [3]. This tension depends on the magnitude of the applied force, which is examined in finite element models [4]. The tension on the pressure side reduces when too much force is used, or completely closes the blood flow in the capillaries, causing hyalinization of the tissue [5,6].

Additionally, fluid from the periodontium is forced into the bone on the pressure side, while on the relaxation side, it flows into the PDL gap [7]. Nerve endings and fibroblasts in the periodontium also respond to the applied force, activating various signaling pathways [3].

Bone remodeling is associated with the activity of osteoblasts and osteoclasts. On the pressure side, there is a three-day apoptosis of osteoblasts, activation of osteocytes, and activation of resorption through an increase in prostaglandins, TNF-alpha, and RANKL [8,9,10,11]. However, on the stretching side, osteocytes are activated in the signaling pathway, resulting in the production of type 1 collagen, osteocalcin, and osteopontin, the number of TNF-beta increases and the concentration of RANKL decreases, causing the formation of new bone tissue and the remodeling of the PDL [12,13,14,15]. This makes the PDL and the surrounding bone form a functional whole.

Procedures such as corticotomy-assisted orthodontic therapy (CAOT) or periodontally accelerated osteogenic orthodontics (PAOO) are intended to reduce bone density and negative stress on the compression side, limiting bone resorption during orthodontic movement or adding bone substitute material so the tooth does not cross the cortical vestibular plate [16,17].

In the literature, there are several studies concerning the impact of these procedures on the efficiency of tooth movement and the risk of complications during orthodontic tooth movement in various planes. To create the most accurate clinical guidelines, it is necessary to gather, systematize, and summarize the available knowledge in this area.

This study aimed to present the effects of additional procedures such as CAOT or PAOO on periodontal tissues during orthodontic treatment, researched and described in scientific publications. The null hypothesis was that they have a beneficial effect on the health of periodontal tissues.

## 2. Methods

This systematic review has been registered in the PROSPERO database under the identification number CRD42024572064.

This study was conducted in accordance with the PRISMA (Preferred Reporting Items for Systematic Reviews and Meta-Analyses) guidelines. The study design was defined in the PICO format:

Population (P): patients with full permanent dentition, both adolescents and adults; Intervention (I): orthodontic treatment with fixed appliances using additional supportive treatments such as CAOT or PAOO.

Comparison (C): assessment of the impact of additional treatments during orthodontic treatment on the remodeling of the alveolar bone and the condition of the periodontium. Result (O): statistically significant/non-significant differences in the condition of the alveolar bone before and after orthodontic treatment.

Electronic databases were searched, including PubMed, Google Scholar, Web of Science EMBASE, and Cochrane Central Register of Controlled Trials, using the following keywords:

- Bone remodeling;
- Bone remodeling and orthodontic treatment;
- Bone remodeling and corticotomy or PAOO or CAOT;
- Corticotomy and orthodontics;
- PAOO and orthodontics;
- Bone remodeling and orthodontics;
- Surgical procedures and orthodontics.

Search filters included the time of the publication of the article, systematic reviews from the last five years, and publications that appeared in English. The information provided in the abstracts of systematic reviews that describe the effects of additional procedures during orthodontic treatment such as CAOT or PAOO on the health of periodontium was analyzed. Articles unrelated to the subject of the planned study and those in which tooth movement acceleration was analyzed were excluded.

For the remaining articles, references were reviewed, and journals such as the *American Journal of Orthodontics*, *Dentofacial Orthopedics*, *International Orthodontics*, *Journal of Clinical Orthodontics*, and *Angle Orthodontist* were manually searched.

### Statistical Analysis

During the data analysis, a significant problem made it impossible to conduct a reliable statistical analysis and assess the diversity of results and heterogeneity of the studies. This problem resulted from the lack of consistency between the results of different studies. Significant differences in methods, patient inclusion criteria, and ways of measuring and classifying outcomes meant that attempts to combine these data could lead to the wrong conclusions. Without uniform and comparable data, the risk of distorting the results of statistical analysis is high, which affects their credibility and scientific value. For this reason, it was decided to base the conclusions on a qualitative study of available data, emphasizing the need to standardize future research for precise and repeatable statistical analysis.

## 3. Results

**Figure 1:**
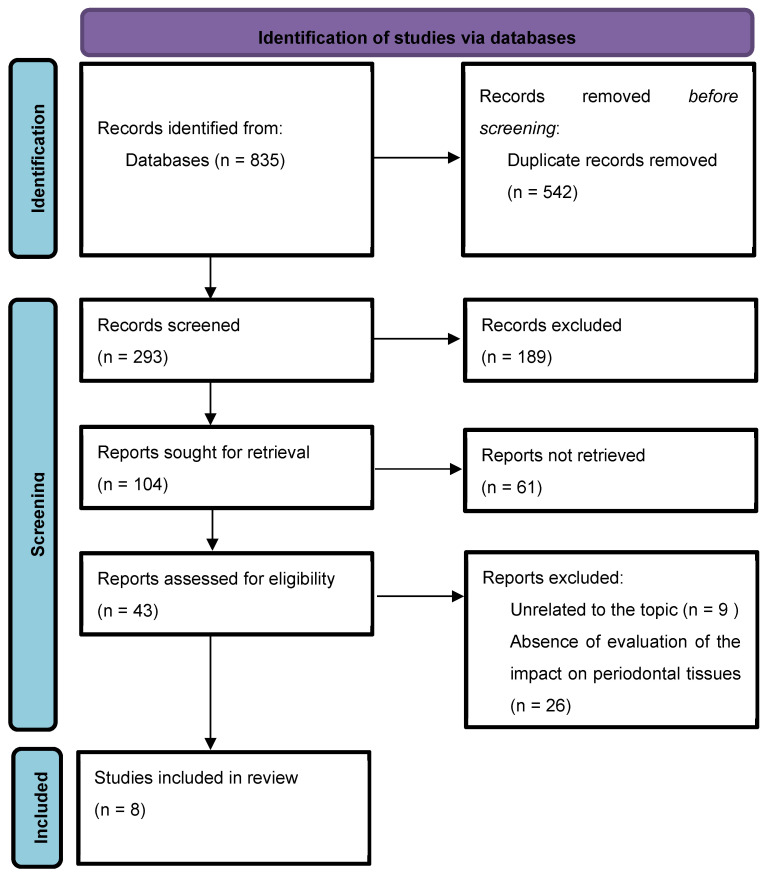
PRISMA flow chart.

The keyword entry yielded about 835 abstracts. A total of 43 articles were initially confirmed as eligible for systematic review and were analyzed in detail. Ultimately, eight articles were selected. The full selection process is shown in Figure 1.

### Risk of Bias

The risk of bias for each study was reported according to the following criteria:

low risk of bias—rated as having a low risk of bias if most of the risks of bias were low;

high risk of bias—rated as high risk of bias if most risks were unclear, moderate, or high [18].

Risk of bias summary is presented in Table 1 and the key for the risk of bias assessment in Table 2:

1. Stefani’s review included only four studies. All studies were described in a way that prevented correct answers to questions related to the risk of bias. This situation resulted in the need to recognize the study as highly biased, which resulted in a high risk of error in the entire review.
2. Dab et al.’s review included 12 studies. Most involved a high degree of methodological heterogeneity, making it difficult to compare and evaluate effectively. The quality of the evidence presented ranged from very low to low. This was influenced by various sources of bias, ranging from potential errors in the assessment of results to methodological problems. There is a shortage of high-quality research on this subject.
3. Guo et al.’s review included two RCTs, three cohort studies, and 21 clinical controlled trials. Fourteen studies were used for qualitative synthesis. The risk of bias in the RCT studies was high and was mainly related to the blinding of investigators and patients. The quality of the cohort studies was moderate and within limits of 15 out of 24 points. Most controlled studies were retrospective, indicating a potentially high risk of bias. The lack of consistent measurement and small sample size represented additional methodological limitations of the included studies. Due to the limited number of included studies, publication bias could not be assessed in each meta-analysis. The overall quality of evidence on the performance assessment is shown. The quality of the evidence was low.
4. Apalinowa’s review included nine studies. All articles presented in the review were considered low-quality because none were described as double-blind and therefore did not exceed three points on the Jadad scale. Two articles that were not defined as randomized received the lowest rating according to Cochrane guidelines. Three studies were considered to have a low risk of bias and on the other hand the remaining six had a high risk of bias due to various issues such as lack of random sequence generation, concealment allocation, or bias due to differences in implementation.
5. Overview Al. Ibrahim included nine studies. This included seven RCTs. Of these studies, two RCTs were classified as low risk of bias, two RCTs were classified as “unclear risk of bias”, while the remaining three were classified as “high risk of bias”. Random sequence generation and blinding of participants were rated as a “high risk of bias” in three RCTs and blinding of outcome assessors was rated as “high risk of bias” in two RCTs. Allocation concealment was unclear in three RCTs. For the only study that included CCT, according to the minor tool, the most problematic domains were the enrollment of consecutive patients and the prospective calculation of the study sample size. The risk of bias was 19/24, indicating moderate quality.
6. Kamal’s review included only five studies. In Kamal’s review, all studies were rated as having a high risk of bias due to various issues such as a lack of random sequence generation, allocation concealment, and bias, due to differences in study subjects’ age, outcome assessment, or subjects withdrawing from the study. Due to small samples and incomplete methodology, there was a common high risk of bias. Furthermore, due to the nature of the intervention, neither the operator nor the patients could be blinded. Other outcomes appropriately considered in the studies remained unclear.
7. The Alsin et al. review consisted of eight studies, including six RCTs and two pilot clinical studies. The risk of bias in the included RCTs can be summarized as follows. Three RCTs were at low risk of bias, and five RCTs were at moderate and high risk of bias. To conceal the allocation, articles with high and moderate risk of bias amounted to 62.5%. Blinding of participants was another problem perceived as a high and moderate risk of bias in 87.5% of included studies. In summary, the risk of error was considered high.
8. Wang et al.’s review consisted of eight studies, including two RCTs and six no-RCTs (including cohort studies). The risk of bias assessment results for the included two RCTSs indicated a higher risk of masking participants and staff (performance bias) and data reporting (reporting and deviation). In addition, six non-RCTs (case–control cohort studies) were assessed using the Newcastle–Ottawa Quality Assessment Scale. Four of the six studies were rated at high risk of bias. The full risk of bias assessment indicated a moderate level of error and a moderate evidentiary quality of the study.

The results of the systematic reviews are presented in Table 3 and below:

- De Stefani et al. showed that corticotomy can only be used in the case of moderate expansion of the dental arch and accompanied by bone augmentation to provide the tooth roots with bone support on all sides; due to the high level of risk of bias, the results are questionable [19].
- Dab et al. concluded that orthodontic treatment with the additional procedure of corticotomy has a low level of evidence of the absence of undesirable side effects of such treatment. According to moderate evidence, an increase in bone density of approximately 7% should be expected in the case of additional augmentation of bone tissue. However, evidence of an increase in the thickness of the atrial lamina by approximately 0.7% has low scientific value [20].
- Gao et al. reported that orthodontic treatment using the PAOO procedure and the application of additional bone substitute materials showed a thicker alveolar ridge and increased bone density after treatment. This SR analyzed studies with a high risk of systematic error, so the evidentiary value of this conclusion is unfortunately low [21].
- Apalimowa et al. compared the results of studies according to which the corticotomy procedure using a flap is more effective than piezocision, although it is more invasive. According to the analysis, corticotomy does not cause major complications such as tooth root resorption, periodontal tissue atrophy, or loss of tooth vitality compared to traditional orthodontic treatment. Still, after treatment, bone density increase can be expected due to the use of bone substitute materials. The results are at a high risk of systematic error, so their reliability must be confirmed [22].
- Al.-Ibrahim et al., although they focused on the acceleration of orthodontic movement, have also shown that during orthodontic treatment with the use of self-ligating brackets with additional piezocision, no gum recession occurred, while with the further use of low-frequency vibrational forces or corticotomy, no deterioration of periodontal tissues was observed. The moderate risk of bias makes the conclusions drawn credible [23].
- Kamal et al. described statistically significant improvement of periodontal tissues due to the use of PAOO; however, the conclusions are subject to a high risk of credibility [24].
- Alsino et al. noted that PAOO leads to an increase in the thickness of the alveolar ridge, although due to the high risk of error, it requires further research [25].
- Wang et al. analyzed studies on the importance of thickening the gingival biotype with tissue augmentation and showed that it might lead to clinical benefits; however, due to the limited number of studies and the moderate risk of bias, it requires further verification [26].

**Table 3:** Results.

LP	Authors of Publication	Additional Procedure	Number of Included Studies	Conclusions
1	De Stefani et al.: Is the corticotomy assisted orthodontic treatment efficient in the expansion of narrow arches in adult patients? [19]	CAOT	4	CAOT with rapid maxillary arch expansion with the ridge augmentation seems to be useful only for moderate dental expansion Uncertain outcomes of that technique
2	Dab S et al.: Short- and long-term potential effects of accelerated osteogenic orthodontic treatment: A systematic review and meta-analysis [20]	CAOT	12	There is a very low to low level of certainty of the effects of CAOT It increases the density of bone after bone grafting The clinical significance of these changes is questionable
3	Gao J et al.: The Significance of Utilizing A Corticotomy on Periodontal and Orthodontic Outcomes: A Systematic Review and Meta-Analysis [21]	PAOO	12	A greater gain of buccal bone thickness and higher postoperative bone density after bone grafting can be achieved
4	Apalimova A et al.: Corticotomy in orthodontic treatment: systematic review [22]	CAOT	9	Bone density may increase as a result of simultaneous placement of bone grafting materials CAOT obtains faster results in comparison to piezocision
5	Al-Ibrahim et al.: The Efficacy of Accelerating Orthodontic Tooth Movement by Combining Self-Ligating Brackets With One or More Acceleration Methods: A Systematic Review [23]	Self-ligating braces and piezocision or low-frequency vibrational forces or flapless corticotomy	9	No gingival recessions No negative effects on periodontal depth No adverse effects on periodontal indices
6	Kamal et al.:Does periodontally accelerated osteogenic orthodontics improve orthodontic treatment outcome? A systematic review and meta-analysis [24]	PAOO	5	The improvement in periodontal health: probing depths and bone density
7	Alsino et al.: The Effectiveness of Periodontally Accelerated Osteogenic Orthodontics (PAOO) in Accelerating Tooth Movement and Supporting Alveolar Bone Thickness During Orthodontic Treatment: A Systematic Review [25]	PAOO	8	It tended to increase the thickness of the alveolar bone
8	Wang et al.: Is periodontal phenotype modification therapy beneficial for patients receiving orthodontic treatment? An American Academy of Periodontology best evidence review [26]	Periodontal phenotype modification therapy with CAOT	8	May provide clinical benefits

## 4. Discussion

During orthodontic treatment, bone resorption always occurs on the side to which the teeth are moved [3]. In the case of extraction treatment, after the retraction of the incisors, the bone on the palatal side of the front teeth thins [2,27,28]. Suppose expansion is necessary for patients after completed growth; in that case, we are dealing with a limited bone envelope and because of this, 20–35% of patients may have problems with recession 2–5 years after orthodontic treatment [26,29,30,31].

Various procedures have been sought and used for years to minimize the effects of expansion in the form of bone dehiscence and gum recession, such as CAOT and PAOO with or without the use of bone substitute materials.

Various methods of maxillary expansion in adult patients are available, such as SARPE or the multi-segment Le Fort I procedure. Still, these are highly invasive procedures that many patients do not accept, as complications such as dehiscence and recession may be observed [19].

This systematic review of other systematic reviews summarizes the latest research findings on the effectiveness of the above procedures. The discrepancy in the methodology of various studies does not allow them to be summarized in a meta-analysis. Many available studies focus on the acceleration of tooth movement and not on protecting bone tissue and tissues adjacent to the tooth [32,33,34]. Nevertheless, appropriate conclusions can be drawn based on some of the studies.

In all reviews, the effectiveness of CAOT and PAOO is unclear. Research evaluation is influenced by the quality of the study and, most often, a high or medium risk of bias. This means that the results should be interpreted with great caution.

Stefani et al. concluded that rapid maxillary expansion combined with corticotomy and additional bone tissue augmentation may be safe and effective in small and medium expansions [19]. In this SR, studies by Hassan, Brugnami, and Sulewska showed CAOT as an effective technique that does not worsen periodontal health after orthodontic treatment [35,36,37]. However, Caccianig’s research combining expansion corticotomy with additional laser therapy proved effective and less invasive than surgical jaw expansion and crossbite correction [38]. Unfortunately, the evidentiary value of these studies is low.

The SR of Dab summarized the results of 12 studies with low or moderate evidence. Additionally, various methods of corticotomy were compared with a complete flap, partial flap, and without a flap, which makes it impossible to interpret the procedure and its effects correctly. The periodontium was assessed according to different indicators, so it was impossible to make a reliable comparison. Nevertheless, it can be assumed that CAOT does not have an adverse effect on the periodontium. In the short-term follow-up, after the addition of bone substitute material, there was an increase in the thickness of the cheekbone after 6 months in favor of corticotomy, but in the long-term follow-up after 6 months post-treatment, the meta-analysis showed a statistically insignificant increase in bone density. Additionally, some studies have observed a significant increase in root resorption [20].

A comparative analysis of CAOT and PAOO with conventional orthodontic treatment performed by Gao showed that after PAOO, a thicker layer of bone remains on the buccal side and is characterized by a higher density after augmentation. Apart from shortening the treatment time, they did not notice the impact of corticotomy on soft tissues, only on the architecture and physiology of bone tissue. The meta-analysis did not show an increase in the width of keratinized tissue. In the case of this study, attention should be drawn to the small sample size, which may have led to skewed results. However, a significant increase in bone thickness of 0.43 mm was demonstrated in the case of the PAOO procedure, which the use of graft material could have influenced. Additionally, PAOO causes the bone morphotype to be transformed into a stronger one, and the addition of bone substitute material may reduce the risk of fenestration and gum recession after orthodontic treatment [21]. The analysis showed a high risk of systematic error, so the conclusions are not binding. It is also worth noting the high heterogeneity of the studies.

Another SR performed by Apalimov assessed that the simultaneous introduction of bone materials can increase bone density, and CAOT produces faster results compared to piezoelectric cutting. She also stated that fenestrations after expansion can be effectively prevented by using bone substitute material. Methods for assessing bone density have varied significantly across studies. However, it turned out that bone density decreased substantially during orthodontic movement in the group treated with corticotomy, both with and without bone graft. Still, it was higher in patients after corticotomy, approximately 26% following treatment. In the control group, it decreased by approximately 17% compared to the state before treatment. The bovine graft was more effective than the bioactive glass in thickening bone structure [22]. Due to the small sample size, the studies had a high risk of bias and low evidentiary value.

Al-Ibrahim analyzed randomized studies and a controlled clinical trial on orthodontic treatment using self-ligating appliances and one of the methods of accelerating tooth movement. His systematic review was characterized by a moderate risk of systematic error and relatively high results credibility. He showed that combining self-ligating brackets from corticotomy with a piezoelectric knife to relieve crowding in both arches did not result in recession or any negative effects on periodontal health [23]. However, only three out of eight analyzed studies examined the effect on the periodontium, unlike others, which mainly examined the effect of the acceleration of tooth movement.

The analysis conducted by Kamal showed a statistically significant positive effect of PAOO on the periodontal condition of patients after orthodontic treatment in the form of a reduction in the depth of gum pockets. However, these results should be interpreted with caution due to the low evidentiary value. Moreover, after orthodontic treatment, due to poor oral hygiene, pockets deepen, unrelated to the orthodontic treatment itself. Bone density may also increase only temporarily; some bone replacement material is resorbed after a year [24].

Alsino summarized the results of eight RCT studies that showed a tendency to increase bone thickness after treatment with PAOO. Two studies have proven the beneficial effect of PAOO on increasing the width and thickness of the alveolar bone. However, further research on a larger group of subjects is necessary to confirm this. Some studies have described that both after corticotomy itself and after PAOO, bone density was reduced at the end of treatment, which is explained by trauma-causing local osteoporosis. On the contrary, other studies have described an increase in bone density at the end of treatment, which may result from incorporating bone substitute material into existing bone. There was no difference in the depth of periodontal pockets between groups [25]. Due to the low value of evidence and the high risk of systematic error, further studies are necessary to confirm or exclude the observed changes in bone and periodontal tissues.

Wang performed an interesting analysis on the modification of the periodontal phenotype with augmentation of bone and/or soft tissue using corticotomy. This analysis shows that bone augmentation combined with corticotomy may bring benefits in increasing the range of motion of the mandibular incisors and their stabilization. Phenotype modification has no specific benefits due to the limited number of studies [26]. The moderate risk of bias makes it difficult to establish these benefits clearly.

Bone remodeling depends not only on the orthodontic movement itself but also on the surgical procedures used. Systemic factors influence bone remodeling. The most studied is the impact of uncontrolled diabetes, which may impair the body’s ability to remodel [39]. Vitamin D also plays an important role in this process. Based on available research, it can be assumed that vitamin D injections may increase the range of tooth movement. Moreover, it can influence os rank/opg by reducing inflammatory cytokines and modifying gene expression [40]. Pregnancy, lactation, and the estrogen cycle change metabolism and may affect preformed bones, but research is limited and concerns animals [41,42]. Psychological stress may also affect bone remodeling [43].

The additional use of lasers may influence the effects of CAOT or PAOO. The evidentiary value of such studies is low, but combining a surgical procedure with lasers may increase the benefits of their separate use [44].

New hopes are associated with the use of cell therapy in bone regeneration. However, it is uncertain whether cells grown in vivo have an advantage over using whole tissues. More detailed research is necessary [45].

The meta-analyses and systematic reviews presented in this publication indicate an increase in bone thickness and density after the use of bone graft material as a part of the PAOO treatment [21,22,46,47]. This procedure is usually used in cases of thin buccal bone where bone graft materials in terms of bone-derived material and bioabsorbable collagen membranes benefit the surrounding soft and hard tissues to transfer the bone from a thin biotype to a more robust type [21,46,48]. In addition, it has been confirmed that bone grafting materials can be used to correct pre-existing bone dehiscences and fenestrations as well as reduce the risk of their occurrence and gingival recession as part of an orthodontic procedure [6,21,49,50].

PAOO offers the benefit of transforming the bone from a thin to a more robust bone morphotype and is indicated in orthodontic situations where there is a concern that orthodontic movement may move the tooth outside the bone envelope [21,48,50]. It should be noted that PAOO therapy requires close cooperation and acceptance of the treatment by the patient, taking into account the patient’s fear and possible postoperative swelling and pain [6,51]. However, the presented results of the meta-analyses should be interpreted with caution due to limitations resulting from the heterogeneity of the included studies, short (3–6 months) follow-up period, and other significant confounding factors regarding the bone grafts and surgical methods used [21,46,47,51]. Before these facilitated orthodontic procedures can be fully utilized in everyday clinical practice, reliable conclusions must be obtained from further well-designed RCTs. Therefore, there is an urgent need to conduct high-quality clinical trials, with particular attention paid to study design, methodology for measuring outcomes, and especially safety and potential adverse effects.

Future well-standardized RCTs are required to minimize study discrepancies with particular attention to sample size and long-term follow-up and a thorough assessment of bone structure after augmentation.

Considering the small sample size and the relatively short duration of the follow-up, future standardized RCTs with large samples are required to explore the effect of augmented corticotomy strategy on the bone density.

## 5. Limitations

The main limitation of this review included systematic reviews written in English within the last five years. This may affect the risk of statistical publication bias. Only those studies that described the effect of additional treatments on periodontal tissues and not on the acceleration of tooth movement were included.

## 6. Conclusions

The above studies show that the validity of CAOT and PAOO treatments remains controversial due to the research’s low evidentiary value. Better results are obtained when combined with tissue augmentation or thickening of the gingival phenotype, rather than stand-alone procedures, and their use to protect periodontal tissues is limited.

## 7. Future Directions

High-quality randomized trials are needed that would additionally condition the obtained measurements of other factors, such as the speed and biomechanics of orthodontic movement, the applied orthodontic force, and the age of patients, to draw reliable conclusions.

## Figures and Tables

**Table 1:** Risk of bias assessment.

	Author of the Review	Number of Studies Included in the Review	Number of Tests		Was the Randomization Scheme Described and Appropriate?		Whether the Study Was Described as Double-Blind		Was the Method Appropriate?		Was the Number of Resignations Described?		Quality		Overall Quality of the Study
			RCT	NRCT	N	IN	N	IN	N	IN	YES	NO	Low	High	
1.	Stefani et al. [19]	4	?	?	4	0	0	4	0	4	0	4	4	0	Low
2.	Dab et al. [20]	12	?	?	?	?	?	?	?	?	?	?	12	0	Low
3.	Guo R et al. [21]	14	2	12	0	2	0	2	1	1	2	0	2	0	Low
4.	Apalinowa et al. [22]	9	7	2	4	5	0	9	?	?	6	3	9	0	Low
5.	Al. Ibrahim et al. [23]	9	7	2	4	3	4	3	5	2	9	0	3	4	Moderate
6.	Kamal et al. [24]	5	2	3	0	5	1	4	3	2	1	4	5	0	Low
7.	Alsino et al. [25]	8	6	2	3	5	1	7	8	0	7	1	5	3	Low
8.	Wang et al. [26]	8	2	6	2	0	0	2	2	0	0	2	2	0	Low

**Table 2:** Risk of bias assessment.

	Author of the Review	Random Sequence Generation		Hide Assignment		Bias Due to Differences in Implementation		Performance Appraisal Bias		Participant Withdrawal Bias		Reporting Bias		Other Sources of Bias		Risk of Bias		Overall Assessment of the Risk of Error
		IN	N	IN	N	IN	N	IN	N	IN	N	IN	N	IN	N	Low	high	
1.	Stefani et al. [19]	0	4	0	4	4	0	4	0	4	0	4	0	4	0	0	4	High
2.	Dab et al. [20]	7	5	10	2	11	1	7	5	3	9	4	8	10	2	2	10	High
3.	Guo et al. [21]	1	1	1	1	2	0	2	0	0	2	0	2	0	2	2	0	High
4.	Apalinowa et al. [22]	7	3	2	7	6	3	4	5	9	0	8	1	0	9	3	6	High
5.	Al. Ibrahim et al. [23]	3	4	3	4	3	4	2	5	0	7	0	7	0	7	4	3	Moderate
6.	Kamal et al. [24]	2	3	2	3	2	3	2	3	4	1	3	2	0	4	2	3	High
7.	Alsino et al. {25]	5	3	?	?	7	1	1	7	?	?	2	6	8	0	3	5	High
8.	Wang et al. [26]	0	2	0	2	2	0	1	1	2	0	0	2	0	2	0	2	Moderate

## Data Availability

The datasets used and/or analyzed in this current study are available from the corresponding author upon reasonable request.

## List of Shortcuts

PRISMA	Preferred Reporting Items for Systematic Reviews and Meta-Analyses
PICO	Population, Intervention, Comparison, Outcome
RCT	Randomized Clinical Trial
CCT	Controlled Clinical Prospective Trial
ROBINS-I	Risk of Bias in Non-randomized Studies of Interventions
QAS	Quality Assessment Scale
CAOT	Corticotomy-assisted orthodontic therapy
PAOO	PeriodontallyAcceleratedOsteogenicOrthodontics
